# Case Report: Primary sporadic intramedullary malignant peripheral nerve sheath tumor with intracranial extension and a subpial nodule suggestive of early dissemination

**DOI:** 10.3389/fonc.2026.1796859

**Published:** 2026-06-29

**Authors:** Fangyu Zhu, Xiyu Zhao, Zhiqiang Ouyang

**Affiliations:** 1 Department of Radiology, Yan’an Hospital Affiliated To Kunming Medical University, Kunming, China; 2 Department of Pathology, Yunnan Provincial Hospital of Chinese Medicine, Kunming, China; 3 Department of Radiology, Yan’an Hospital of Kunming City (Yan’an Hospital Affiliated to Kunming Medical University), Kunming, China

**Keywords:** case report, cervical spinal cord, dedifferentiation, intramedullary MPNST, neuraxial dissemination, tumor recurrence

## Abstract

**Background:**

Intramedullary malignant peripheral nerve sheath tumors (MPNSTs) are exceptionally rare, particularly in sporadic cases without neurofibromatosis type 1 (NF−1) or prior radiotherapy.

**Case presentation:**

A 22-year-old male presented with progressive left-sided weakness, gait imbalance, and sensory disturbance. MRI revealed an intramedullary C2 mass with intracranial extension and a small dorsal subpial enhancing nodule at C5, raising concern for early neuraxial dissemination. Gross total resection confirmed MPNST. Recurrence developed 18 months later with left cerebellar extension and immunophenotypic dedifferentiation. The patient subsequently experienced rapid clinical deterioration and died 14 months after recurrence.

**Conclusion:**

This case highlights the highly aggressive biological behavior of sporadic intramedullary MPNST, including rapid progression despite gross total resection. Early recognition of atypical imaging features suggestive of dissemination and consideration of multimodal treatment strategies may be important for optimizing surveillance and management.

## Introduction

Malignant peripheral nerve sheath tumors (MPNSTs) are rare and aggressive soft-tissue sarcomas commonly associated with neurofibromatosis type 1 (NF1) or prior radiation exposure (1–3). They typically occur in the trunk, extremities, and head and neck regions, and are associated with high recurrence and metastatic potential (1, 2). Primary spinal cord tumors account for approximately 2–4% of central nervous system neoplasms, among which intramedullary tumors are predominantly astrocytomas and ependymomas (4). Intramedullary spinal involvement by MPNST is exceedingly rare, with only a limited number of sporadic, NF1-associated, post-radiation, and malignant transformation-associated cases reported in the literature to date (**Table 1**) (4–7). Reported patients commonly present with progressive weakness, sensory disturbances, pain, and gait impairment consistent with spinal cord myelopathy, while treatment has primarily consisted of maximal surgical resection with variable use of adjuvant radiotherapy or chemotherapy (4–7).

**Table 1:** Reported cases of intramedullary spinal malignant peripheral nerve sheath tumors in the literature.

Author/year	Age/sex	Type	NF−1	Prior RT	Primary/metastatic	Level	Intracranial extension/dissemination	Recurrence	Treatment	Outcome
Paolini et al., 2006 (5)	50/M	Post-radiation	No	Yes	Primary	C4–C5	None reported	NR	STR; No RT	Died 9 mo, pneumonia-related complications
Marton et al., 2011 (4)	56/M	Sporadic primary	No	No	Primary	C2–C3	None reported	None at 9 mo	GTR + chemotherapy (ifosfamide)	No evidence of recurrence at 9 mo
Newell et al., 2021 (6)	35/F	NF1-associated; post-radiation	Yes	Yes	Indeterminate	C7–T1	Presumed Leptomeningeal dissemination (cerebral cortex and cauda equina)	Radiologic progression at 1 yr	STR + RT	DOD 1 yr
Albrefkany et al., 2025 (7)	53/M	Malignant transformation from schwannoma	No	No	Primary	T11	None reported	NR	STR → GTR + planned RT	NR
Present Case (this report)	22/M	Sporadic primary	No	No	Primary	C2–Medulla	Early subpial dissemination suspected; intracranial extension at recurrence	Yes	GTR(1st and 2nd operation); No RT	Died 14 mo after recurrence

Type categories included sporadic primary, NF1-associated, post-radiation, and malignant transformation-associated tumors.

NF−1, neurofibromatosis type 1; RT, radiotherapy; STR, subtotal resection; GTR, gross total resection; NR, not reported; DOD, died of disease.

Among reported cases, sporadic intramedullary MPNSTs without NF1 or prior radiotherapy are particularly rare.² The present case is notable for cervicomedullary involvement with intracranial extension at recurrence, suspected early subpial dissemination at presentation, hemorrhagic progression, and immunophenotypic progression at recurrence, suggesting aggressive neuraxial behavior. This report further expands the limited literature on sporadic intramedullary spinal MPNSTs.

## Case presentation

### Clinical presentation

A 22-year-old male presented with a four-month history of progressive left-sided weakness, gait imbalance, limb paresthesia, and neck pain. He had no NF−1 stigmata, no history of radiotherapy, and no spinal trauma.

Neurological examination revealed no meningeal irritation signs. The patient demonstrated left-sided hemiparesis with Medical Research Council grade 2+/5 strength, decreased muscle tone, impaired hand grip, and weakened ankle dorsiflexion. Sensory examination revealed impaired superficial and deep sensation over the left hemibody with preserved cortical sensory modalities. Left-sided dysmetria and gait ataxia were present. Bilateral Babinski signs were elicited. Cranial nerve examination was largely intact without dysarthria, dysphagia, or lower cranial nerve dysfunction. No bowel or bladder dysfunction was identified. Functional status was consistent with modified McCormick grade III.

### Initial imaging findings

Preoperative contrast-enhanced sagittal T1-weighted MRI showed an intramedullary tumor centered at the C2 level extending cranially into the medulla (**Figure 1A**). A small dorsal subpial enhancing nodule at C5 raised concern for early neuraxial dissemination (**Figure 1B**). Preoperative differential considerations included high-grade glioma, ependymoma, astrocytoma, and other intramedullary spinal neoplasms. Initial imaging evaluation consisted primarily of contrast-enhanced MRI, and no additional advanced preoperative imaging modalities were performed.

**Figure 1:**
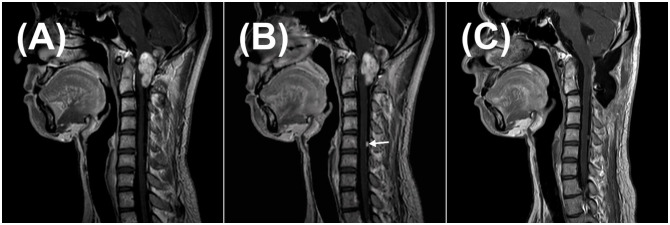
Preoperative and postoperative contrast-enhanced sagittal T1-weighted MRI of the cervical spinal cord. **(A)** Preoperative contrast-enhanced sagittal T1-weighted MRI demonstrating an intramedullary mass centered at the C2 level with cranial extension into the medulla. **(B)** Preoperative contrast-enhanced sagittal T1-weighted MRI showing a small dorsal subpial enhancing nodule at the C5 level (arrow). **(C)** Postoperative contrast-enhanced sagittal T1-weighted MRI confirming gross total resection of the intramedullary lesion without residual enhancement.

### First operation and pathology

The patient underwent posterior suboccipital craniotomy with upper cervical laminectomy for tumor resection. Under microscopic visualization, internal tumor debulking was first performed using ultrasonic aspiration, followed by careful circumferential microsurgical dissection from the surrounding parenchyma. Gross total resection was achieved. Histopathological examination demonstrated a malignant spindle cell neoplasm with patchy S−100 positivity, diffuse CD34 expression and a Ki−67 index of 20–30%, consistent with intramedullary MPNST (**Figures 2A–C**).

**Figure 2:**
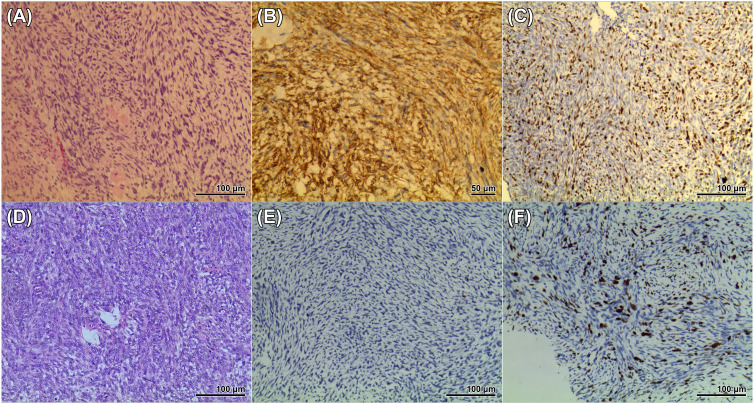
Histopathological features of the primary and recurrent tumors. **(A)** Hematoxylin and eosin (H&E) staining of the primary tumor showing densely cellular spindle-shaped tumor cells arranged in interlacing fascicles. **(B)** Immunohistochemical staining for S-100 protein in the primary tumor demonstrating diffuse cytoplasmic positivity. **(C)** Ki-67 immunostaining of the primary tumor demonstrating a labeling index of 20–30%. **(D)** H&E staining of the recurrent tumor revealing similar spindle-cell morphology. **(E)** S-100 immunostaining of the recurrent tumor showing markedly reduced S-100 expression. **(F)** Ki-67 immunostaining of the recurrent tumor demonstrating a labeling index of 20–30%. Scale bars are provided for reference.

### Postoperative course

Postoperative MRI confirmed no residual tumor (**Figure 1C**). Although adjuvant radiotherapy was strongly recommended postoperatively, the patient declined further treatment because of financial limitations.

### Recurrence

Eighteen months later, the patient presented with worsening gait disturbance accompanied by headache, dizziness, nausea, and vomiting. There were no signs of meningeal irritation. The patient was alert but required assistance for ambulation. Mild weakness of shoulder shrug and neck rotation was present. Motor examination demonstrated mild residual left-sided weakness with Medical Research Council grade 4+/5 strength and decreased muscle tone, whereas strength and tone in the right extremities were preserved. Sensory examination of the upper extremities was symmetric and intact, with preserved cortical sensory modalities and normal coordination testing. No pathological reflexes were elicited. Cranial nerve examination was largely intact without dysarthria, dysphagia, or other lower cranial nerve dysfunction. No bowel or bladder dysfunction was identified. Functional status at recurrence remained consistent with modified McCormick grade III.

MRI revealed a recurrent mass extending upward into the left cerebellum with obstructive hydrocephalus (**Figures 3A, B**). Both MRI and CT demonstrated intratumoral mixed-age hemorrhage within the recurrent lesion (**Figures 3C, D**). Mildly enhancing postoperative cervical cystic cavities were present along the cervical spinal cord, favoring reactive postoperative changes rather than dissemination.

**Figure 3:**
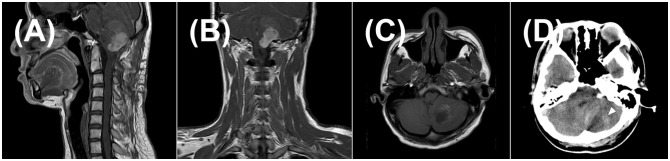
Imaging findings of tumor recurrence. **(A)** Contrast-enhanced sagittal T1-weighted MRI demonstrating recurrent tumor extending from the cervical spinal cord to the medulla and posterior fossa. **(B)** Contrast-enhanced coronal T1-weighted MRI showing extension of the lesion into the left cerebellar hemisphere. **(C)** Axial T1-weighted MRI revealing focal areas of relatively high signal intensity within the tumor, suggestive of intratumoral hemorrhage. **(D)** Axial CT scan demonstrating patchy hyperdense areas within the lesion (arrow), corresponding to intratumoral hemorrhage.

### Second operation

Repeat surgery was performed through the previous posterior occipital approach with extension of the craniotomy. Internal decompression was performed using ultrasonic aspiration, followed by microsurgical circumferential dissection to achieve gross total resection. Histopathological analysis demonstrated higher nuclear atypia, increased necrosis, markedly reduced S−100 expression, persistent CD34 positivity, and a Ki−67 index of 20–30%, indicating immunophenotypic dedifferentiation (**Figures 2D–F**). Adjuvant radiotherapy was again recommended after reoperation but was declined because of financial constraints. Despite repeat surgical management, the disease progressed rapidly, and the patient died 14 months after recurrence. A timeline summarizing the patient’s clinical course is shown in **Figure 4**.

**Figure 4:**
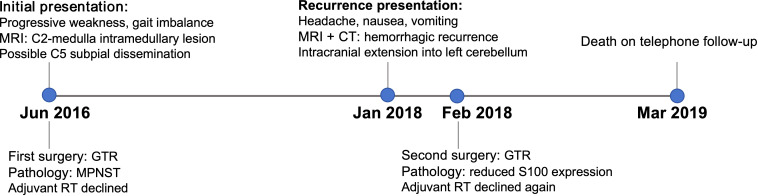
Timeline of the patient’s clinical course. Summary of symptom onset, imaging findings, surgical interventions, pathological progression, treatment decisions, recurrence, and clinical outcome from initial presentation in June 2016 to death in March 2019.

## Discussion

Intramedullary MPNSTs are exceptionally uncommon (4, 5) and represent a diagnostic challenge due to their rarity and non-specific MRI features, which can mimic other intramedullary neoplasms such as ependymomas or astrocytomas (2, 4). The differential diagnosis of intramedullary spinal tumors includes astrocytoma, ependymoma, hemangioblastoma, and less commonly primary spinal sarcomas or malignant nerve sheath tumors. Astrocytomas and ependymomas represent the most common intramedullary neoplasms in adults and frequently demonstrate overlapping MRI characteristics, including cord expansion and contrast enhancement (4, 8). Although MPNSTs may radiologically resemble other intramedullary tumors or peripheral nerve sheath neoplasms, Although these findings are not specific for intramedullary MPNST, the presence of aggressive features such as rapid neurological deterioration, hemorrhagic change, infiltrative growth, and possible leptomeningeal dissemination may suggest a high-grade malignant process (2, 9). Definitive diagnosis therefore relies on histopathological and immunohistochemical evaluation in conjunction with imaging findings.

A particularly noteworthy finding was the small dorsal subpial enhancing nodule at C5. Although pathological confirmation was unavailable, its morphology and location along the pial surface raise a plausible suspicion of early neuraxial dissemination. Prior reports have demonstrated that intradural and intramedullary MPNSTs possess the potential for leptomeningeal and cerebrospinal fluid dissemination, including cases with diffuse spinal and intracranial seeding (10, 11). In this context, the present lesion may represent an early radiological manifestation of CSF pathway spread. Subpial lesions may therefore serve as potential early indicators of leptomeningeal dissemination in aggressive spinal cord malignancies.

Postoperative cervical CSF-like cystic cavities with mild rim enhancement created additional diagnostic uncertainty. Their CSF-like signal, smooth margins, and temporal association with surgery were more compatible with reactive postoperative changes than dissemination. Nonetheless, continued vigilance is warranted, as MPNSTs have the potential to disseminate via cerebrospinal fluid pathways.

At recurrence, the tumor demonstrated intracranial extension into the cerebellum and intratumoral mixed-age hemorrhage. Intratumoral hemorrhage has been reported in MPNSTs (12, 13) and is believed to result from tumor-associated vascular fragility, angiogenesis, and necrotic degeneration. In our case, the mixed-age hemorrhagic components likely reflect repeated micro-bleeding and progressive vascular instability during malignant progression. Mixed-age hemorrhage is a recognized imaging marker of aggressive tumor behavior and aligns with the dedifferentiated histopathology observed at recurrence.

The recurrent tumor also exhibited immunophenotypic dedifferentiation, including markedly reduced S-100 expression, increased necrosis, and an elevated Ki-67 index. Loss of S−100 expression is commonly observed in high-grade or dedifferentiated MPNSTs (2) and may correlate with more aggressive clinical behavior and poorer outcomes.

Management of intramedullary MPNST remains particularly challenging because of the tumor’s infiltrative behavior, eloquent spinal cord location, and high risk of recurrence. In the present case, both procedures were planned through posterior midline approaches to maximize tumor exposure while minimizing additional neurological injury. The first operation consisted of posterior suboccipital craniotomy with upper cervical laminectomy for decompression of the cervicomedullary junction and maximal safe tumor resection. At recurrence, repeat surgery through the previous posterior occipital corridor with extension of the craniotomy allowed resection of the recurrent lesion extending into the posterior fossa and left cerebellum. Because the lesion involved the cervicomedullary junction, the infiltrative nature of the tumor and distortion of normal anatomy increased the risk of injury to residual functional long tracts and lower cranial nerve pathways. Therefore, microsurgical resection was performed using internal debulking with ultrasonic aspiration followed by cautious circumferential dissection to minimize traction on adjacent neural tissue while achieving gross total resection. Similar operative strategies incorporating neuromonitoring and maximal safe resection have been described in previously reported intramedullary MPNST cases (6, 7).

Although gross total resection remains the cornerstone of treatment for MPNSTs, recurrence rates remain high even after aggressive surgery (1, 2). Prior reports of intramedullary spinal MPNSTs have used combinations of subtotal or gross total resection with adjuvant radiotherapy or chemotherapy, although long-term outcomes have generally remained unfavorable because of the aggressive biological behavior and recurrence potential of these tumors (**Table 1**) (4–7). In the current case, adjuvant radiotherapy was strongly recommended after both surgeries because of the aggressive pathological features and suspected early dissemination; however, treatment was declined because of financial limitations. This represents an important therapeutic limitation, as the absence of adjuvant radiotherapy may have reduced local disease control and allowed progression of potential microscopic or disseminated disease.

The role of adjuvant therapy in MPNST remains incompletely defined, particularly for rare intramedullary lesions. Several retrospective studies suggest that postoperative radiotherapy may improve local control, especially in high-grade tumors or cases with close surgical margins, although its effect on overall survival remains controversial (1, 2, 14). Chemotherapy has shown variable activity, with sporadic MPNSTs appearing to demonstrate somewhat greater responsiveness than NF1-associated tumors in prospective studies (15). However, the present case illustrates that even sporadic intramedullary MPNSTs may exhibit highly aggressive biological behavior and poor clinical outcomes despite gross total resection. Emerging targeted therapies, including inhibition of the mTOR and EGFR pathways and multikinase inhibitors such as pazopanib, have demonstrated preliminary activity in preclinical or early-phase studies, although their clinical efficacy remains uncertain (2, 16). Given the highly aggressive biological behavior demonstrated in this case, including suspected early dissemination, hemorrhagic progression, intracranial extension, and immunophenotypic dedifferentiation, multimodal treatment strategies and close postoperative surveillance may be particularly important.

To our knowledge, this appears to be the first reported sporadic intramedullary MPNST demonstrating both intracranial extension and a radiologically identified subpial lesion suggestive of early dissemination. Early recognition of atypical imaging features, such as subpial nodules and mixed-age hemorrhage, may facilitate earlier diagnosis and closer follow-up. Given the overlapping imaging characteristics with other aggressive intramedullary tumors, definitive diagnosis requires multimodal assessment incorporating radiological, histopathological, and immunohistochemical evaluation (2, 4).

## Conclusion

Sporadic intramedullary MPNSTs may demonstrate highly aggressive biological behavior, including intracranial extension, early neuraxial dissemination, hemorrhagic progression, recurrence, and immunophenotypic dedifferentiation. Despite gross total resection, the present case illustrates that even sporadic intramedullary MPNSTs may exhibit rapid progression and poor clinical outcomes, highlighting the limitations of surgery alone in aggressive disease. Early recognition of atypical imaging findings and consideration of multimodal treatment strategies may be important for optimizing disease surveillance and management.

## Data Availability

The original contributions presented in the study are included in the article/supplementary material. Further inquiries can be directed to the corresponding author/s.
